# Ilio-Iliac Arteriovenous Fistula Secondary to a Ruptured Right Common Iliac Artery Aneurysm and Anomalous Anatomy of Inferior Vena Cava Resulting in an Arteriovenous Shunt Formation with Right-sided Cardiac Failure: A Case Report

**DOI:** 10.70352/scrj.cr.24-0094

**Published:** 2025-01-31

**Authors:** Yuki Shirai, Aya Saito, Chiharu Tanaka, Yuki Moriyama, Yuya Ito, Kazuyuki Ishibashi, Noboru Motomura

**Affiliations:** 1 Department of Cardiovascular Surgery, Toho University Sakura Medical Center, Sakura, Chiba, Japan; 2 Department of Cardiovascular Surgery, Yokohama City University Hospital, Yokohama, Kanagawa, Japan; 3 Department of Gastroenterological Surgery, Toho University Sakura Medical Center, Sakura, Chiba, Japan; 4 Wakasa Clinic Hibarigaoka, Nishitokyo, Tokyo, Japan; 5 Department of Cardiovascular Surgery Ship International Hospital, Dhaka, Bangladesh

**Keywords:** common iliac artery aneurysm, ilio-iliac arteriovenous fistula, right-sided cardiac failure, inferior vena cava anomalies, arteriovenous shunt formation

## Abstract

**INTRODUCTION:**

An ilio-iliac arteriovenous fistula (IIAVF) secondary to the rupture of a common iliac artery aneurysm (CIAA) is rare. Sudden arteriovenous shunting and subsequent fistula enlargement can result in acute cardiac failure. Immediate diagnosis and treatment are required; however, the clinical symptoms differ from those of a free wall rupture of an aortic aneurysm, making a quick diagnosis difficult. Thus, we reported the case of a patient with severe right-sided cardiac failure, due to an arteriovenous shunt formation secondary to an IIAVF who underwent an artificial blood vessel replacement with favorable results.

**CASE PRESENTATION:**

A 71-year-old male patient presented to our hospital with polypnea and palpitations. Initial early-phase computed tomography (CT) revealed a 60-mm-in-diameter right CIAA and an inferior vena cava (IVC) dilatation. Severe congestive heart failure, due to an arteriovenous shunt formation secondary to an IIAVF was diagnosed. The massive shunt of blood flowed from the right common iliac artery (CIA) to the right common iliac vein (CIV). He underwent an emergency open abdominal aortic replacement. The IVC ran anomalously anterior to the giant right CIAA and strongly adhered to the IVC and right CIV. Two guidewires were inserted from the bilateral femoral veins into the IVC intraoperatively. Moreover, an occlusion balloon was inserted into the right CIV. Thus, bleeding from the fistula was well-controlled by the time of aneurysm opening. The proximal side of the artificial graft was anastomosed to the abdominal aorta, while the right and left peripheral branches of the prosthesis were anastomosed to the right external iliac artery and left CIA, respectively.

**CONCLUSIONS:**

We reported the case of a giant right CIAA that directly created a shunt into the right CIV. Contrast-enhanced CT is a useful method for confirming the working diagnosis of an IIAVF. In particular, in cases of IVC anomalies or strong perivenous tissue adhesions, bleeding can be controlled using devices, such as occlusion balloons and a meticulous surgical plan.

## Abbreviations

AAA
: abdominal aortic aneurysm
AVF
: arteriovenous fistula
CIA
: common iliac artery
CIAA
: common iliac artery aneurysm
CIV
: common iliac vein
CT
: computed tomography
CVP
: central venous pressure
EIV
: external iliac vein
IIAVF
: ilio-iliac arteriovenous fistula
IVC
: inferior vena cava
PG
: pressure gradient
TR
: tricuspid regurgitation

## INTRODUCTION

The incidence of inferior vena cava (IVC) perforation of abdominal aortic aneurysms (AAAs) has been reported to be approximately 2.2%, and the exact incidence of ilio-iliac arteriovenous fistula (IIAVF), an arteriovenous fistula (AVF) between the common iliac artery (CIA) and common iliac vein (CIV), secondary to the rupture of a CIA aneurysm (CIAA) is unknown, as only a few cases have been reported.^1)^ An IIAVF can cause a sudden arteriovenous shunt and acute cardiac failure.^2)^ As the symptoms of this condition differ from those of a free wall rupture of an aortic aneurysm, determining the pathophysiology thereof is challenging. Moreover, an AVF that is diagnosed late, resulting in shock has a mortality rate of 50%. However, providing treatment prior to the onset of shock can decrease the mortality rate to 25%.^3)^

Consequently, we report the case of a patient with severe congestive heart failure, due to an arteriovenous shunt formation secondary to an IIAVF who underwent an artificial graft replacement with favorable results.

## CASE PRESENTATION

### History and examination

A 71-year-old male patient suddenly experienced polypnea and palpitations, which resulted in an inability to climb stairs. Thus, he consulted with his local doctor. The patient had a history of hyperuricemia, chronic kidney disease, and ureterolithiasis; however, he was not taking any medications. He had smoked approximately 30 cigarettes per day for the past 50 years. Owing to pain and swelling of the right thigh, the working diagnosis of a pulmonary thromboembolism was made. Therefore, he was transferred to the Cardiology Department of Toho University Sakura Medical Center.

### Preoperative investigations

Echocardiography and contrast-enhanced computed tomography (CT) scan revealed the absence of a deep vein thrombosis and pulmonary thromboembolism; nonetheless, a giant right CIAA was found; and the patient was referred to us for surgical intervention.

Chest radiography revealed that the pulmonary artery was dilated, as evidenced by the hilum convergence sign. Transthoracic echocardiography showed right ventricular dilatation, tricuspid regurgitation (TR) with a pressure gradient (PG) of 44 mm Hg, and dilatation of the IVC to 21 mm in diameter. Laboratory blood tests revealed an elevated brain natriuretic peptide of 452 pg/mL. Consequently, the patient was diagnosed with acute right-sided cardiac failure and pulmonary hypertension.

**Fig. 1:**
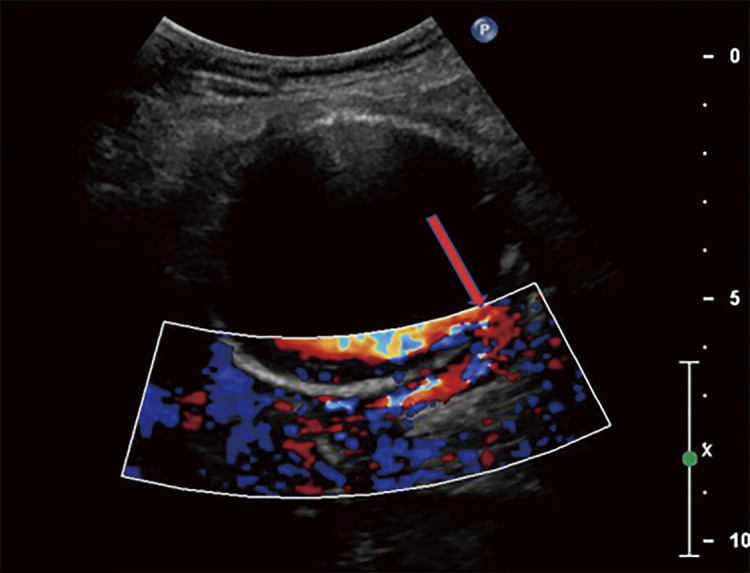
Abdominal ultrasonography revealing a right CIAA compressing the right CIV. Depicted are the absence of an intravenous thrombus and the presence of a 64 × 59 mm right CIAA, with shunt flow to the adjacent right CIV, where the proximal aspect is dilated (arrow). CIAA, common iliac artery aneurysm; CIV, common iliac vein

**Fig. 2:**
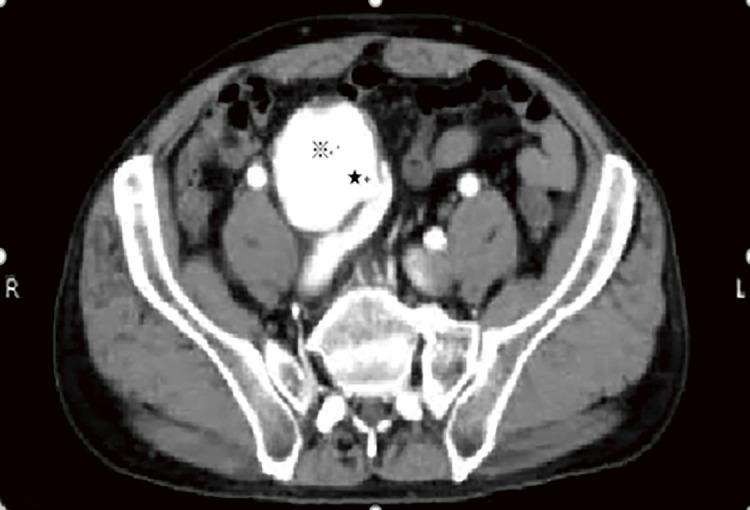
Early-phase contrast-enhanced computed tomography is shown. Contrast-enhanced computed tomography reveals a 60-mm-in-diameter right CIAA and contrast effects (※), from the right CIA to the right CIV (★), confirming the diagnosis of an IIAVF. The IVC runs anterior to the right CIAA and is dilated at the proximal aspect. Because contrast medium outflow into the CIV has been observed in the early phase, a large shunt is thought to have formed. CIAA, common iliac artery aneurysm; CIV, common iliac vein; CIA, common iliac artery; IVC, inferior vena cava; IIAVF, ilio-iliac arteriovenous fistula

**Fig. 3:**
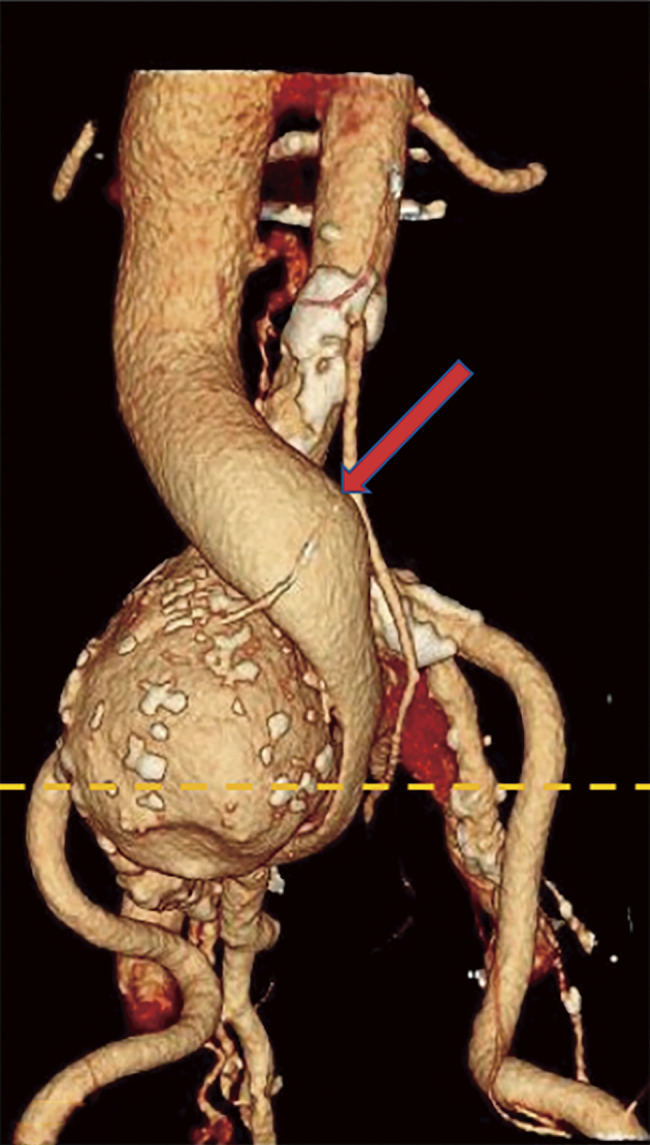
A three-dimensional constructed image, created using contrast-enhanced computed tomography is shown. The yellow dotted line in this image corresponds to the cross-section shown in **Fig. 2**. The IVC runs anterior to the right CIAA and is dilated on the proximal side (red arrow). CIAA, common iliac artery aneurysm; IVC, inferior vena cava

Abdominal ultrasonography revealed the absence of an intravenous thrombus and the presence of a right CIAA, with shunt flow to the adjacent right CIV, where the proximal aspect was dilated (**Fig. 1**). CT confirmed a 60 mm in diameter right CIAA and contrast effects, from the right CIA to the right CIV in the early phase, confirming the diagnosis of an IIAVF. The IVC ran anterior to the right CIAA and was dilated on the proximal aspect. (**Figs. 2** and **3**).

### Preoperative clinical course

The IIAVF had been diagnosed on the fourth day of the onset of right-sided cardiac failure. Based on the patient’s age, in the absence of a critical medical background, we planned an emergency open abdominal aortic replacement, with a Y-graft vascular prosthesis, as opposed to a stent-graft insertion. Preoperative blood tests revealed normal coagulability; and an occlusion balloon was prepared, should massive intraoperative bleeding occur.

### Surgery

**Fig. 4:**
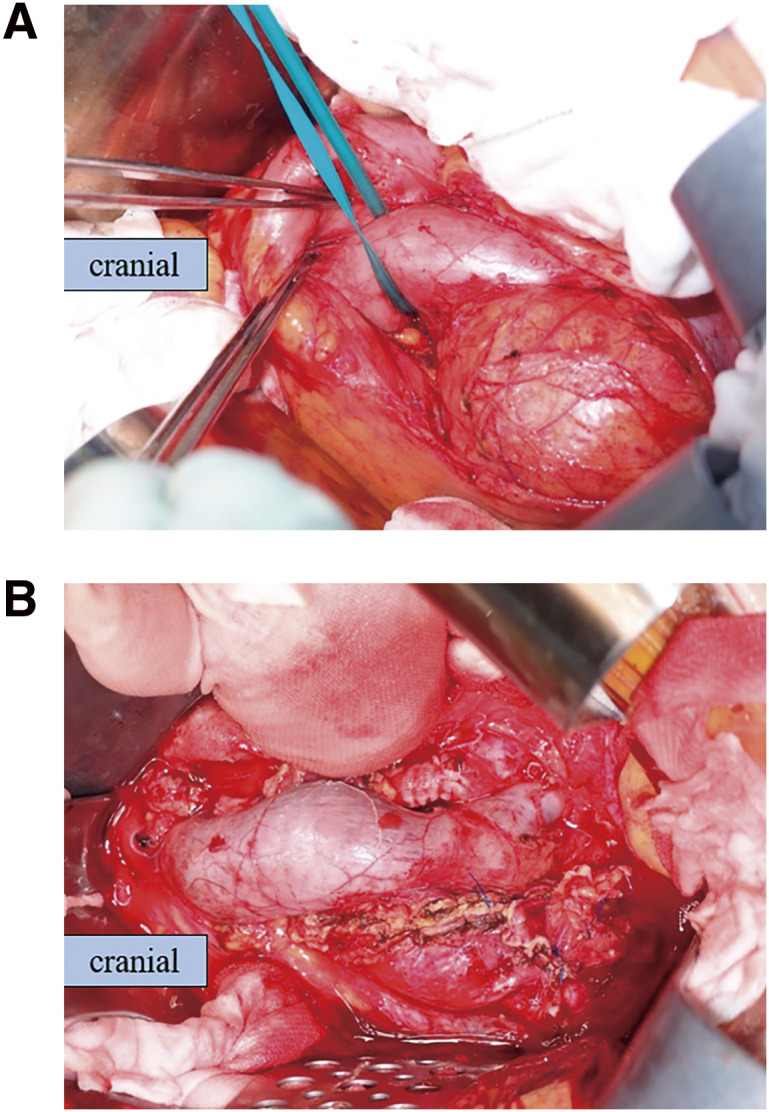
Intraoperative images. (**A**) The IVC travels anterior to the right CIAA, as in the computed tomographic view; and adhesions are observed. No rupture into the abdominal cavity or retroperitoneal space is observed. (**B**) The fistula has been closed directly from within the aneurysm. Only minimal thrombus formation within the aneurysm is observed. Bleeding from the fistula has been well controlled. CIAA, common iliac artery aneurysm; IVC, inferior vena cava

A midline incision was made in the abdomen under general anesthesia into the abdominal aorta. The right internal and external iliac arteries, left CIA and CIV, and IVC were taped. A guidewire was inserted through the left and right femoral veins. The right external iliac vein (EIV) could not be dissected from the surrounding tissue, owing to severe adhesions; and an occlusion balloon was introduced into the right EIV from the right femoral vein (**Fig. 4A**).

**Fig. 5:**
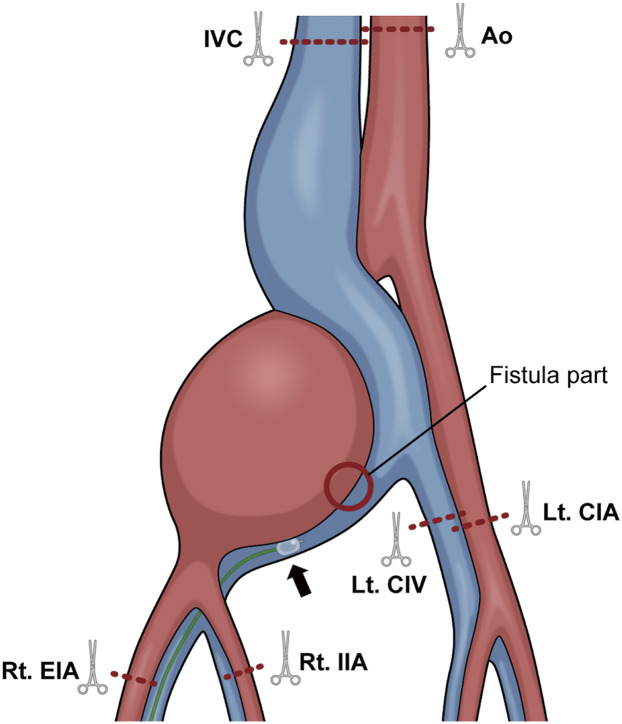
Points where clamping forceps and occlusion balloons were used. The abdominal aorta and IVC, left CIV, left CIA, right EIA, and right IIA were clamped, respectively (dotted red line). An occlusion balloon was inflated on the distal side of the right CIV and the bleeding was controlled (black arrow). IVC, inferior vena cava; CIV, common iliac vein; CIA, common iliac artery; EIA, external iliac artery; IIA, internal iliac artery

**Fig. 6:**
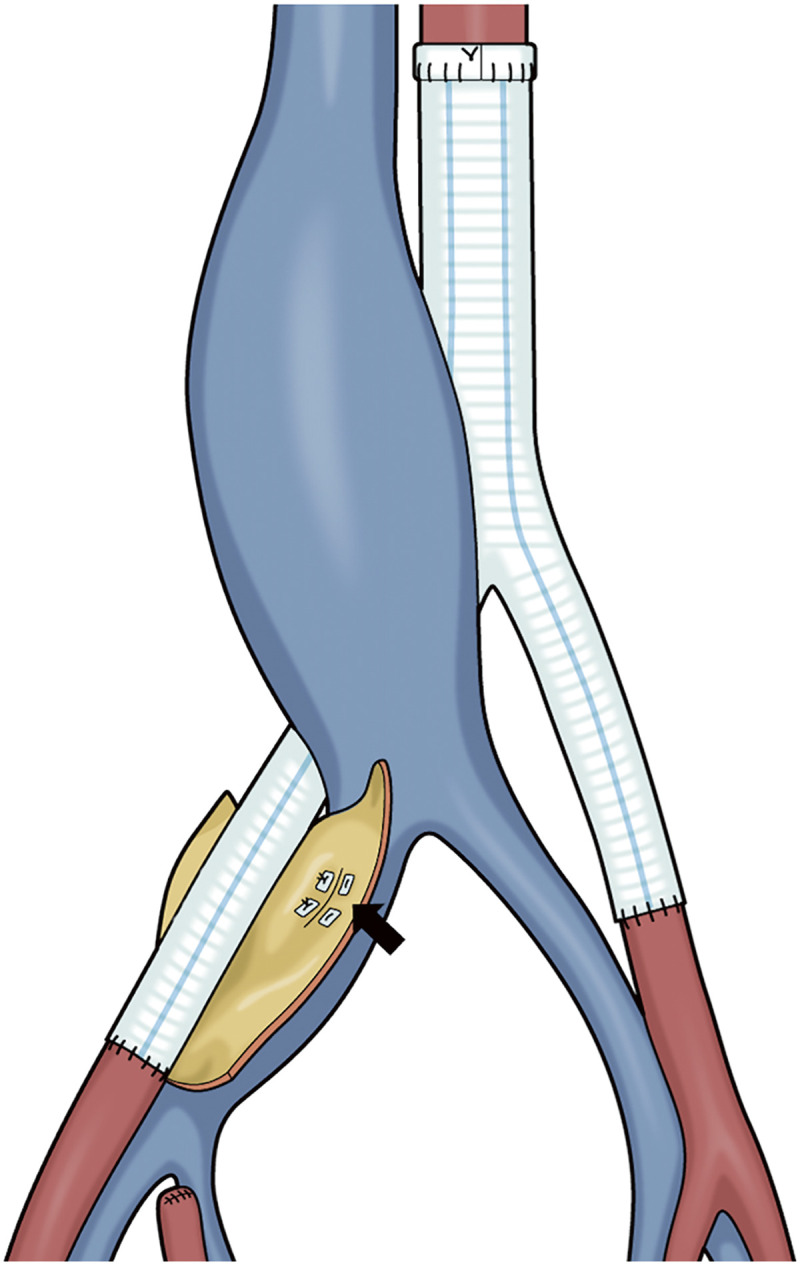
Outline of the surgical procedure. We used a Y-shaped vascular prosthesis (Gelsoft 14 × 7 mm) for the artificial vessels. The bifurcation of the right external iliac artery is aneurysmal; therefore, a distal anastomosis has been performed with the right external iliac artery. On the left side, a distal anastomosis with the CIA has been performed. The IVC fistula was closed directly (black arrow). CIA, common iliac artery; IVC, inferior vena cava

After the incision of the aneurysm, the fistula was identified and closed. The right internal iliac artery was directly closed by a 4-0 monofilament suture into dissected sections. All taped vessels were clamped, the occlusion balloon was inflated in the right CIV, and the bleeding was well controlled; hence, the guidewire inserted from the left femoral vein was not used (**Fig. 5**). Thereafter, the proximal end of the Y-shaped vascular prosthesis (Gelsoft 14 × 7 mm SO-631407P; Terumo, Tokyo, Japan) was anastomosed to the infrarenal abdominal aorta, and the distal side was anastomosed to the right external iliac artery and left CIA (**Figs. 4B** and **6**). After declamping the Y-graft, the central venous pressure (CVP) decreased from 30 to 10 mm Hg.

### Postoperative clinical course

Postoperative chest radiography revealed improved permeability of the bilateral lung fields and reduced pulmonary artery opacity. Contrast-enhanced CT was performed on the sixth postoperative day, confirming the disappearance of the IIAVF. No anomalies were observed at the anastomosis site of the Y-graft. Echocardiography revealed a reduction in the IVC diameter and TR PG, from 21 to 8 mm in diameter and from 44 to 21 mm Hg, respectively.

The patient experienced no complications and was discharged on the 15th postoperative day.

## DISCUSSION

### Principal findings

A 71-year-old male patient developed severe right-sided cardiac failure, due to an arteriovenous shunt formation. This occurred secondary to an IIAVF, as a complication of a ruptured CIAA. He underwent an artificial blood vessel replacement with favorable results. Initially, the working diagnosis of a pulmonary thromboembolism from a venous thrombus was made on ultrasonography. However, subsequent contrast-enhanced CT revealed an IIAVF as the definitive diagnosis.

### Method of diagnosis

For an AAA to form a fistula with the IVC at rupture is rare, with an estimated prevalence of 3%–4% for AAAs, respectively.^4)^ The classic triad of an abdominal AVF is a high-output cardiac failure, a pulsatile abdominal mass accompanied by a thrill or bruit, unilateral lower-extremity ischemia, or venous enlargement. However, all these signs have been found in approximately 50%–80% of cases.^5,6)^ Furthermore, some cases of AVF are asymptomatic.^7)^ Before the CT era, 75% of cases of AVF could not be diagnosed preoperatively.^8)^ Thus, both ultrasound and contrast-enhanced CT are critical for the prompt and accurate diagnosis of AVF.^9,10)^

### Did the anomalous course of the IVC cause AVF?

In the present case, the IVC that ran anterior to the right CIA was regarded as a preaortic IVC.^11)^

The IVC anomaly is relatively rare, with a prevalence of 0.5%–1%.^12)^ Nevertheless, preaortic IVCs are usually asymptomatic, with little clinical significance.^13)^ Regarding AAAs and other retroperitoneal organs, this anomaly complicates the surgical view, making surgery more difficult. Whether this preaortic IVC directly contributes to the growth of AAAs is unclear. However, the structural proximity thereof to the AAA may cause severe adhesions or contribute to the formation of an AVF. From another perspective, this may be one of the factors preventing rupture into the retroperitoneal space or intraperitoneal cavity.

### Surgical strategy

In this case, the principal concern was controlling retrograde bleeding from the AVF when the aneurysm was incised, as has been reported by Gyoten et al.^14)^ In their report, they used two balloon catheters to control the bleeding resulting from the large fistula while preventing a pulmonary embolism due to an atheroma or air.^14)^

Conversely, the venous tissue around the fistula is dilated and friable because of a large amount of shunt flow and may potentially be at risk for intimal injury or vascular lacerations when using an occlusion balloon.

As a recommendation, the fistula in the IVC should be closed from the lumen side of the aneurysm, which includes the IVC wall. In particular, if the IVC wall could be dissected, direct closure with venous tissue alone is likely to result in uncontrolled bleeding, after declamping.^15)^ Additionally, if the caliber of the fistula is large, patch plasty of the vein and artificial vascular replacement of the aorta should be considered.^14)^ However, in this case, based on preoperative CT and intraoperative findings, we determined that the fistula in the IVC could be closed directly. Uemura et al.^16)^ have observed that in cases of a large fistula with poor arteriovenous wall conditions that make direct closure or patch formation difficult, the use of a trimmed greater omentum to plug the fistula in the aneurysm could be another option.

## CONCLUSION

We described the case of a patient who developed severe right-sided cardiac failure due to an arteriovenous shunt formation. This occurred secondary to an IIAVF, as a complication of a ruptured right CIAA. After a meticulous evaluation to accurately assess the preoperative condition, venous flow, and fistula site, an emergency open abdominal aortic replacement and direct fistula closure using an occlusion balloon were performed. Our study highlighted that prompt and in-depth assessments are essential for successful surgical procedures.

## ACKNOWLEDGMENTS

We would like to thank Editage (www.editage.com) for English language editing. We are also grateful to Medical Fig. (https://medicalfig.medicaleducation.co.jp/home) for their help with the illustrations.

## DECLARATIONS

### Funding

None.

### Authors’ contributions

YS, AS, and NM designed the research.

YS wrote the initial draft of the paper.

AS, CT, and NM supervised this study and revised the paper.

AS, YM, YI, KI, and NM were involved in treating the patient.

All authors read and approved the final manuscript.

All authors consent to take responsibility for all aspects of the research.

### Availability of data and materials

The data supporting the conclusions of this article are included within the article.

### Ethics approval and consent to participate

This work does not require ethical considerations or approval.

### Consent for publication

Informed consent was obtained from the patient described in this article.

### Competing interests

The authors declare that they have no competing interests.
